# Prospective comparison of static versus dynamic images in abdominal ultrasound education - a randomised controlled trial

**DOI:** 10.1186/s12909-025-07711-9

**Published:** 2025-07-23

**Authors:** Johannes Matthias Weimer, Michael Eigenseher, Simon Alexander Stiehl, Dieter Nürnberg, Michael Ludwig, Marie Stäuber, Andreas Weimer, Roman Kloeckner, Klaus Dirks, Liv Lorenz, Holger Buggenhangen, Julia-Weinmann-Menke, Daniel Merkel

**Affiliations:** 1 Rudolf Frey Learning Clinic, University Medical Center of the Johannes Gutenberg University Mainz, Langenbeckstraße 1, 55131 Mainz, Germany; 2 Department of Internal Medicine I, University Medical Center of the Johannes Gutenberg University Mainz, Mainz, Germany; 3 Immanuel Klinik Rüdersdorf, University Hospital of the Brandenburg Medical School, Rüdersdorf b, Berlin, Germany; 4 Brandenburg Institute for Clinical Ultrasound (BIKUS), Brandenburg Medical School Theodor Fontane (MHB), Neuruppin, Germany; 5 Department of Internal Medicine I, Hospital of the German Armed Forces Berlin, Berlin, Germany; 6 Clinic for Trauma and Reconstructive Surgery, University Clinic Heidelberg, 69118 Heidelberg, Germany; 7 Institute of Interventional Radiology, University Hospital Schleswig-Holstein - Campus Lübeck, Lübeck, Germany; 8 Medical Clinic II, University Hospital Würzburg, Würzburg, Germany; 9 Department of Radiation Oncology and Radiotherapy, University Medical Center of the Johannes Gutenberg University Mainz, Mainz, Germany

**Keywords:** Ultrasound education, Static images, Dynamic images, Static vs. dynamic, Digital education, Still images, Motion images

## Abstract

**Introduction:**

In medical ultrasound education, static and dynamic images help learners to understand sonoanatomical and sonopathological findings. However, there is currently no clear evidence or recommendations from professional associations regarding which presentation format is more effective for developing ultrasound competencies. This prospective, randomised, controlled study aimed to investigate the impact of static versus dynamic ultrasound images on learners’ acquisition of theoretical competencies in abdominal sonography.

**Methods:**

Participants in certified ultrasound courses were randomised into two groups following an introductory session on ultrasound basics. Separately, both groups completed training covering normal findings and pathologies of the gallbladder, the liver and the pancreas. The study group underwent a digital training session (18 min total) for each of the three topics using dynamic images (video clips) while the control group received the same training session using static images. After the training, participants of both groups completed an online multiple-choice theory test, consisting of 54 questions with 4 answer options per question.

**Results:**

A total of 145 datasets (69 control group, 76 study group) were included in the analysis. The study group achieved significantly higher overall theory test scores (*p* = 0.001) and performed significantly better in the total score of pathology findings (*p* < 0.001). No significant differences were observed in the total score of normal findings (*p* = 0.08). Multivariate regression analysis identified “group allocation dynamic,” “experience with > 30 ultrasound examinations,” and “employment in internal medicine” as significant positive predictors (*p* < 0.01) of theory test performance.

**Conclusion:**

Dynamic images in ultrasound education improve comprehension of pathological findings over static images. These insights should inform the development and adaptation of future training programs and educational materials to enhance the quality of ultrasound education and diagnostic accuracy.

**Supplementary Information:**

The online version contains supplementary material available at 10.1186/s12909-025-07711-9.

## Introduction

### Background

Ultrasound diagnostics have become an indispensable tool in modern medicine, playing a central role in diagnosis and therapeutic decision-making across various medical specialties [1]. The continuous and unlimited real-time visualisation of organs and structures provide additional diagnostic value compared to other imaging techniques, establishing its high relevance in addressing specific diagnostic questions [1]. Considering its pivotal role, a comprehensive education in ultrasound diagnostics is essential for aspiring physicians. Currently, this education typically involves attending (certified) training courses and supervised clinical practice as part of medical education and professional development [2–4]. Professional societies issue recommendations for ultrasound training at both the undergraduate and postgraduate levels, addressing aspects such as the timing and scope of training, teaching methods and materials and the qualifications of instructors and training institutions [2, 5–8].

Despite these guidelines, the teaching methods employed in ultrasound training vary widely, particularly regarding the use of didactic materials and the presentation of sonographic images and findings [2, 5–7, 9–12]. Trainees are often exposed to both static and dynamic ultrasound images to develop a fundamental understanding of sonoanatomy and sonopathology [9, 13, 14].

### Research problem and question

Although the presentation of images is a cruical component of ultrasound education, there are no clear, evidence-based recommendations on whether static or dynamic images are more effective for teaching theoretical and practical competencies. Previous research in this field primarily focused on the general effectiveness of digital teaching methods [12, 15–17].

Insights from cognitive science suggest that both static and dynamic images—used individually or in combination—can support learning, but their specific effects seem to depend on the context and task [18–21]. A multimedia approach may offer advantages in general learning scenarios [20, 22, 23]. For instance, in psychomotor training for dental students, participants using dynamic videos to learn origami folding techniques demonstrated faster and more accurate replication, likely due to a better intuitive understanding of movement sequences. Conversely, those using static images with audio instructions required more time, but achieved deeper cognitive processing [24]. The literature consistently highlights the benefits of both well-prepared educational films and images [20, 21, 25–28].

Research specifically addressing the advantages and disadvantages of static versus dynamic images in ultrasound education remains scarce. While the benefits of real-time imaging are often emphasised [29], there are currently no evidence-based guidelines on the optimal format (static vs. dynamic) for presenting findings in ultrasound education [2]. This lack of standardization and the existing heterogeneity in training practices raise the question of which type of image presentation most effectively facilitates the acquisition of theoretical knowledge (e.g., understanding normal findings and pathologies) in ultrasound training. This study aims to address this gap by investigating the impact of static and dynamic images on the development of theoretical competencies among ultrasound learners participating in certified abdominal sonography courses. It is hypothesized that dynamic images, with their real-time representation and clinical relevance, may provide superior learning effects compared to static images. Static images, however, might offer unique advantages for detailed analysis of anatomical structures and measurements.

By systematically analysing the effects of both presentation formats on learners’ understanding and application of ultrasound diagnostics, this study seeks to provide evidence-based recommendations for the design of future training programs. Ultimately, this could enhance the quality of ultrasound education, improve patient safety, and support more effective clinical decision-making.

## Material and methodology

### Study design, participant recruitment, and inclusion criteria

**Fig. 1:**
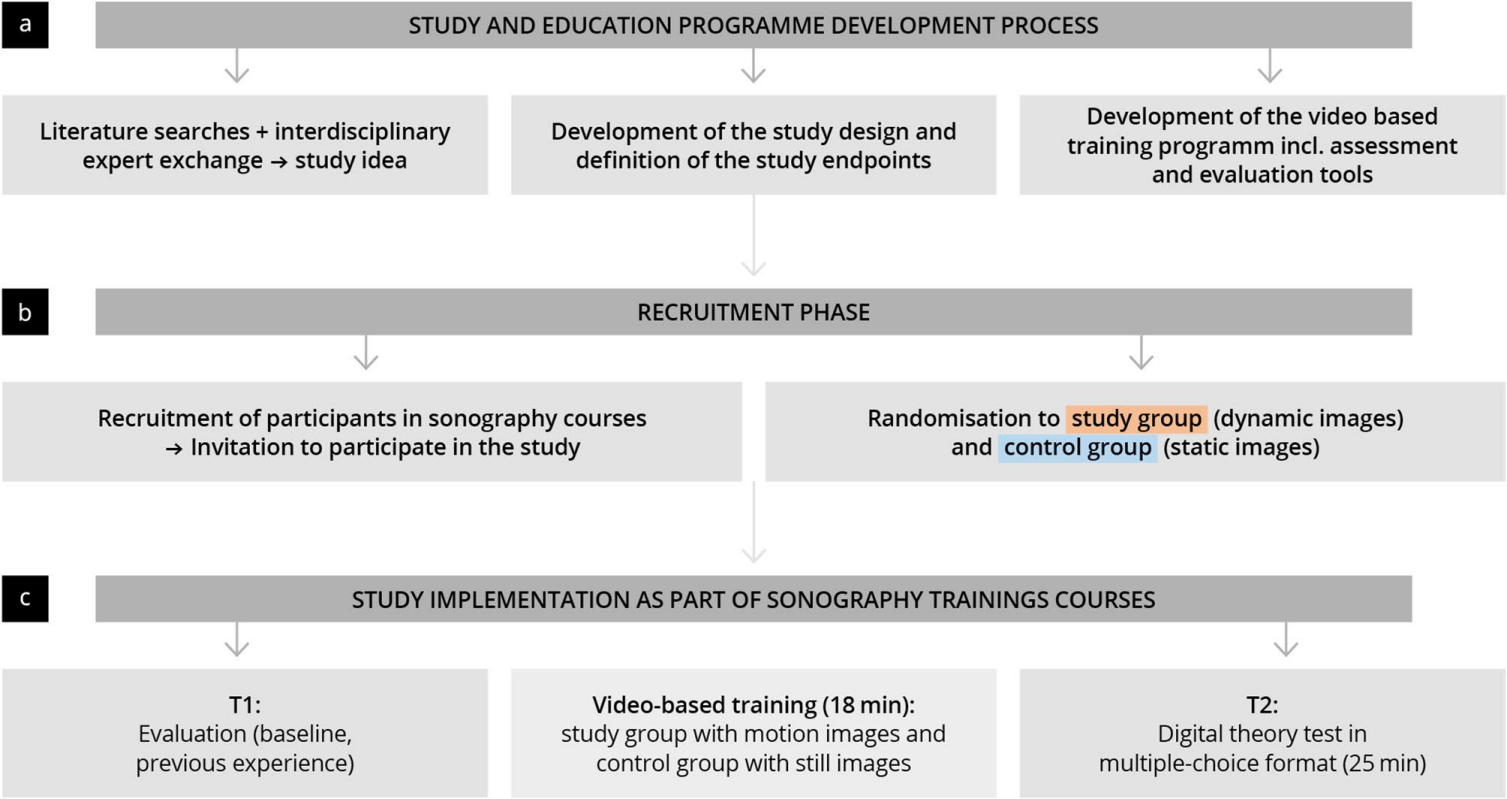
Illustration of the study process, including recruitment, materials, methodology, and measurement timeline

This prospective, controlled, randomized study was conducted between December 2022 and November 2023 as part of certified basic ultrasound courses by the German Society for Ultrasound in Medicine (DEGUM) (see Fig. 1) [30]. Following a 90-minute theoretical introduction covering the basics of ultrasound and image formation, participants were randomly assigned to a control group (video training using static images) or a study group (video training using dynamic images).

Participants were separated into two distinct rooms to avoid potential interference. Both groups received a standardised video training session that presented ultrasound normal findings and pathologies, either as static images (control group) or as dynamic images (study group). The training focused on three topics—gallbladder, liver, and pancreas—lasting a total of 18 min. Immediately after the training, all participants completed a digital theory test consisting of 54 multiple-choice questions (maximum duration: 25 min) and answered additional baseline characteristic questions.

Inclusion criteria were consent to participate, completion of the video training, and full completion of the theory test. The primary endpoint was the competence level, measured by performance in the theory test. Secondary endpoints included the analysis of potential influencing factors.

The study protocol was reviewed and approved by the Ethics Committee of the Medical University Theodor Fontane Brandenburg under the number E-01-20220427 (May 26, 2022).

### Training and theory test

The video training for both groups was identical in duration, spoken text, and displayed captions, differing only in the format of the sonographic findings. In the control group, the ultrasound findings were demonstrated exclusively with static images, while in the study group, only dynamic images were used to present the same ultrasound findings. The training content covered three key topics, each lasting six minutes. The first topic focused on the gallbladder, addressing both normal findings and pathologies, such as gallstones, sludge, and cholecystitis. The second topic dealt with the liver, covering normal findings and various pathologies, including hyperechoic, hypoechoic, and isoechoic lesions. Finally, the third topic explored the pancreas, discussing its normal findings and pathologies related to the head, body, tail, and associated lesions. The training was delivered in a plenary format using a projector and audio system. All sonographic findings were sourced and curated from an online teaching platform [31].

**Fig. 2:**
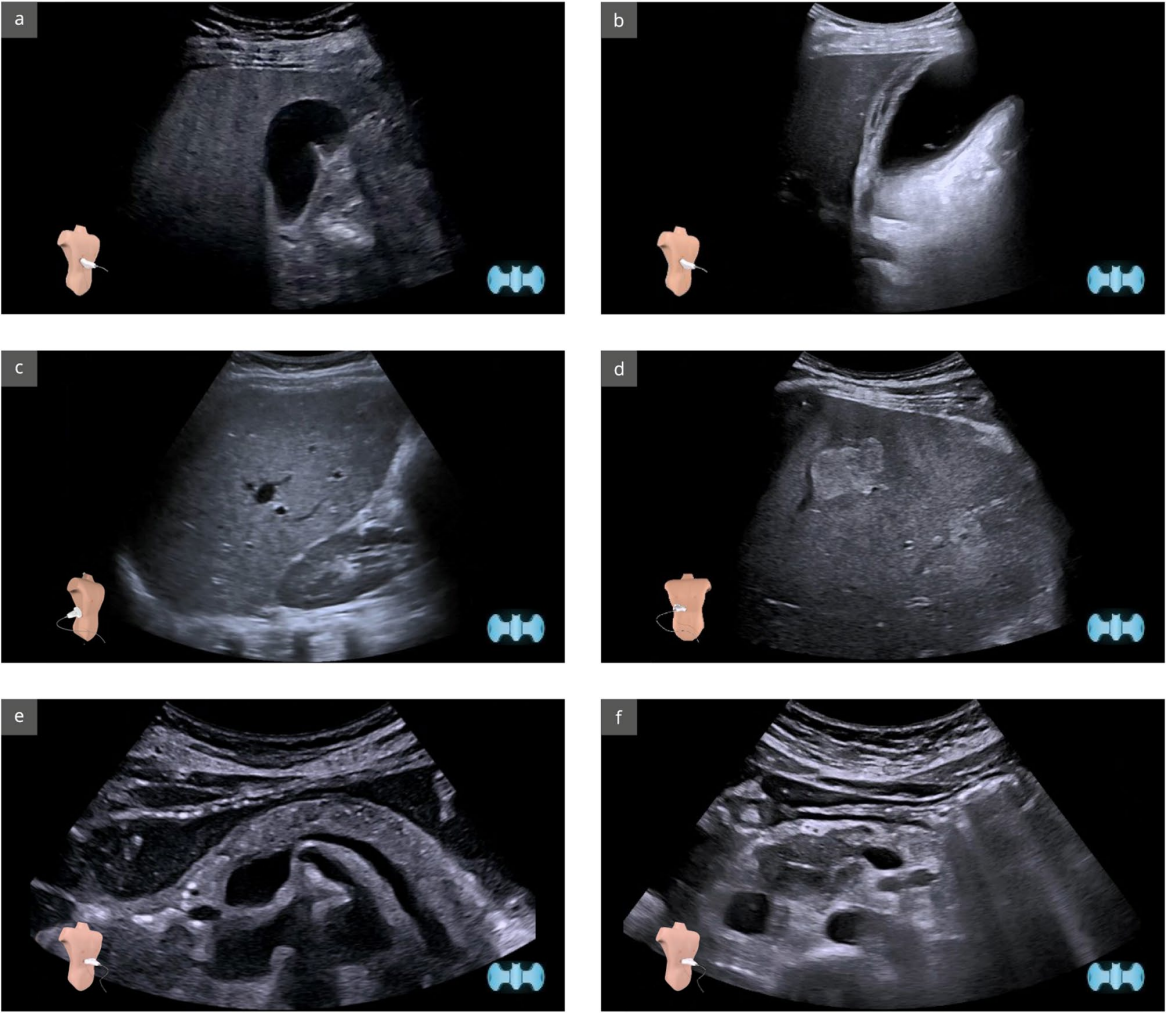
Sample question of the online theory test on the topics (**a**) gallbladder normal finding and (**b**) pathology cholecystitis; (**c**) liver normal finding and (**d**) pathology haemangioma; (**e**) pancreas normal finding and (f) head tumour pathology

Immediately after the training, participants completed a digital theory test in an online multiple-choice format [32]. The test comprised 54 questions (16 for the gallbladder, 20 for the liver, and 18 for the pancreas), each with four answer options, which were always identical for each complex (for example questions/findings see Fig. 2 + Supplement 1). Participants completed the test after scanning a QR code using smartphones or laptops. Each question had a maximum time limit of 30 s.

An interdisciplinary team of ultrasound experts and educators developed the video training and theory test. To ensure content validity, the materials were pretested in a proof-of-concept evaluation by five ultrasound experts (certified as DEGUM Level II or III). At the end of the test, participants answered questions on baseline characteristics like professional position, prior ultrasound experience, specialty, and current employment role.

### Statistical analysis

Data collection was carried out using the survey and test tool Survio (Survio ^®^, Czech Republic). All data were saved with Microsoft Excel. All statistical analyses were performed in Rstudio (Rstudio Team [2020]. Rstudio: Integrated Development for R. Rstudio, PBC, http://www.rstudio.com, last accessed on 20 06 2024) with R 4.0.3 (A Language and Environment for Statistical Computing, R Foundation for Statistical Computing, http://www.R-project.org; last accessed on 20 06 2024). Where possible, a main scale score was made from the average of the subscale scores. Binary and categorical baseline variables are given as absolute numbers and percentages. Continuous data are given as median and interquartile range (IQR) or as mean and standard deviation (SD). Categorical variables were compared using a chi-squared test and continuous variables using a t-test or the Mann-Whitney U test. These tests were also used to calculate the influence of the factors on the objective test results anf for subgroup analysis. In addition, parametric (Anova) or non-parametric (Kruskall-Wallis) analyses of variance were calculated and further explored with pairwise post-hoc tests (either t-test or Mann-Whitney U test). Multivariate linear regression models were constructed to compare the influence of individual factors on the test results. In the multivariate linear regression analysis in relation to the results of the theory tests, group allocation (static vs. dynamic), prior ultrasound experience (> 30 independent examinations), qualification level (student/resident vs. specialist/senior physician), specialty, and workplace (i.e., clinical setting) were defined as possible influencing factors. *P*-values < 0.05 were considered statistically significant. For this study, a power analysis was performed to determine the sample size required to detect a statistically significant effect. Based on an expected effect size of 0.6, a significance level of 0.05, and a desired power of 0.90, the calculated sample size was set at 120 participants (60 in each group).

## Results

### Baseline characteristics of the study and control group

**Fig. 3:**
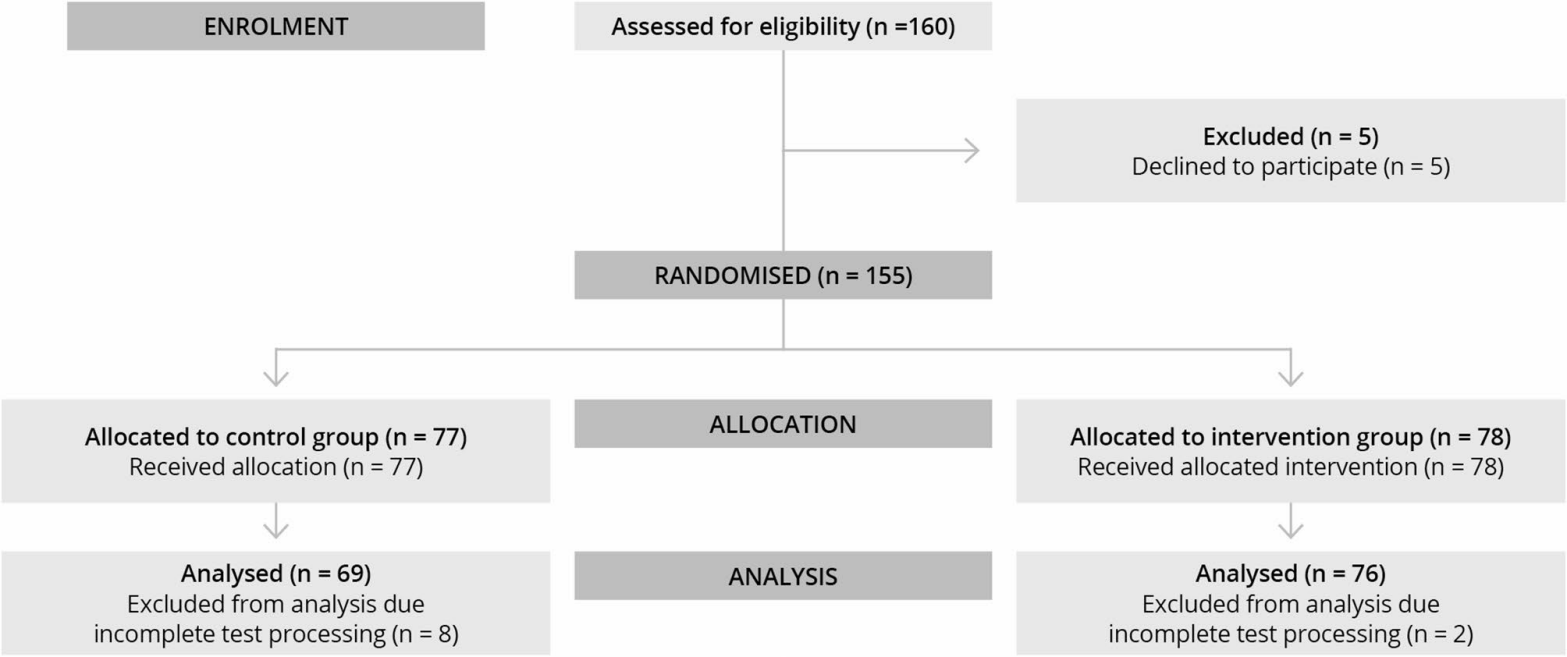
Flow diagram showing participant recruitment and data analysis according to CONSORT guidelines [30]

A total of 145 datasets (see CONSORT Fig. 3 and Supplement 2) were included in the statistical analysis (*n* = 76 in the control group, *n* = 69 in the study group). Both groups exhibited similar demographic profiles regarding qualifications, professional roles, and prior experience. However, there was a significant difference in the participants’ “current specialty” between the two groups. In the study group, a higher proportion of participants were employed in internal medicine (55% vs. 43%), whereas more participants in the control group worked in general medicine (20% vs. 6%). A majority of participants in both groups were residents (study: 68%, control: 63%) and worked in clinical settings (study: 78%, control: 62%). Approximately half of the participants in each group reported to have conducted fewer than 30 independent ultrasound examinations.

### Theory test results

**Fig. 4:**
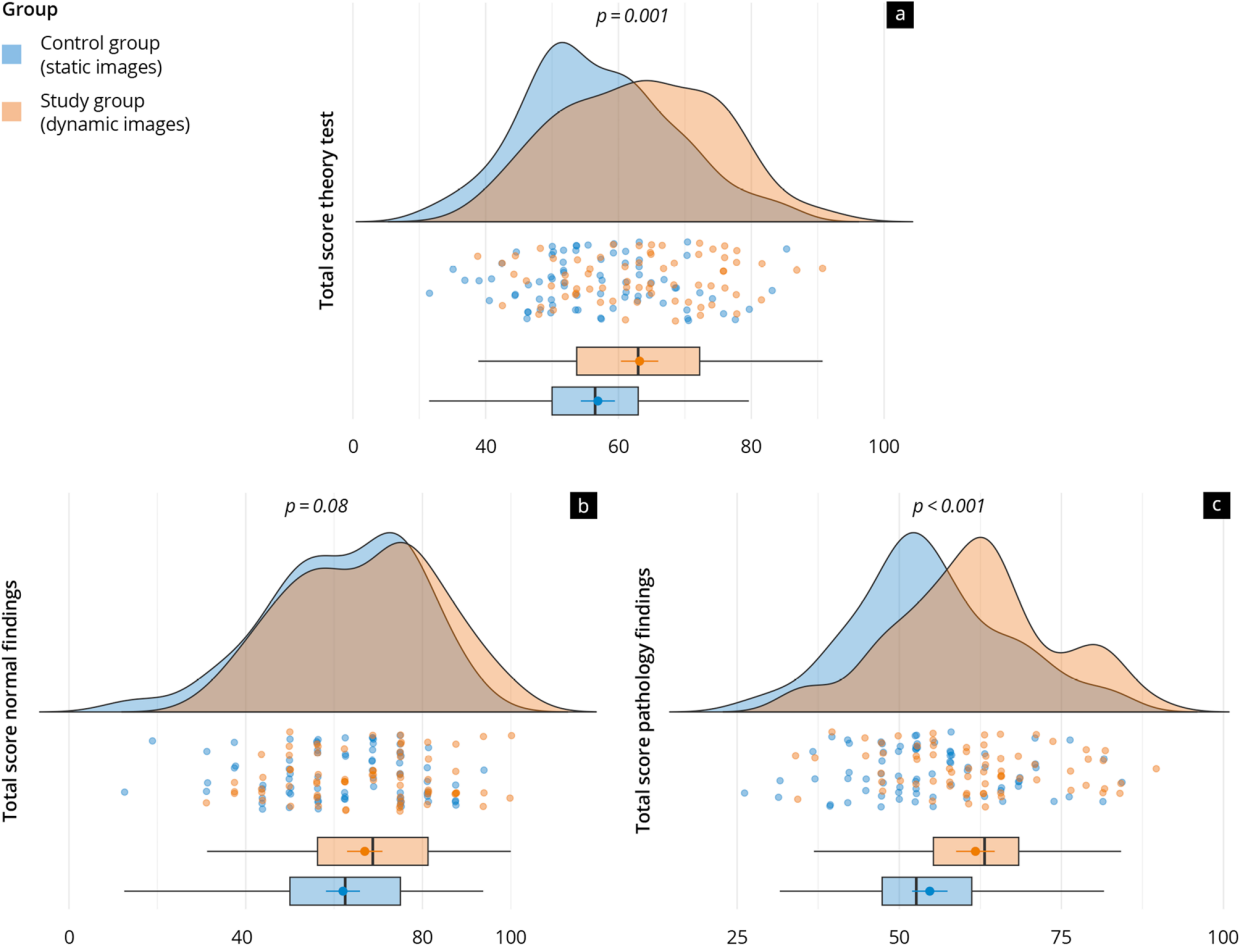
Raincloud plot illustration of the results of the theory test of the control group (‘static’) and study group (‘dynamic’) in (**a**) the overall value, and the competence areas (**b**) ‘normal findings’ and (**c**) ‘pathology findings’. The results are presented as a percentage

**Fig. 5:**
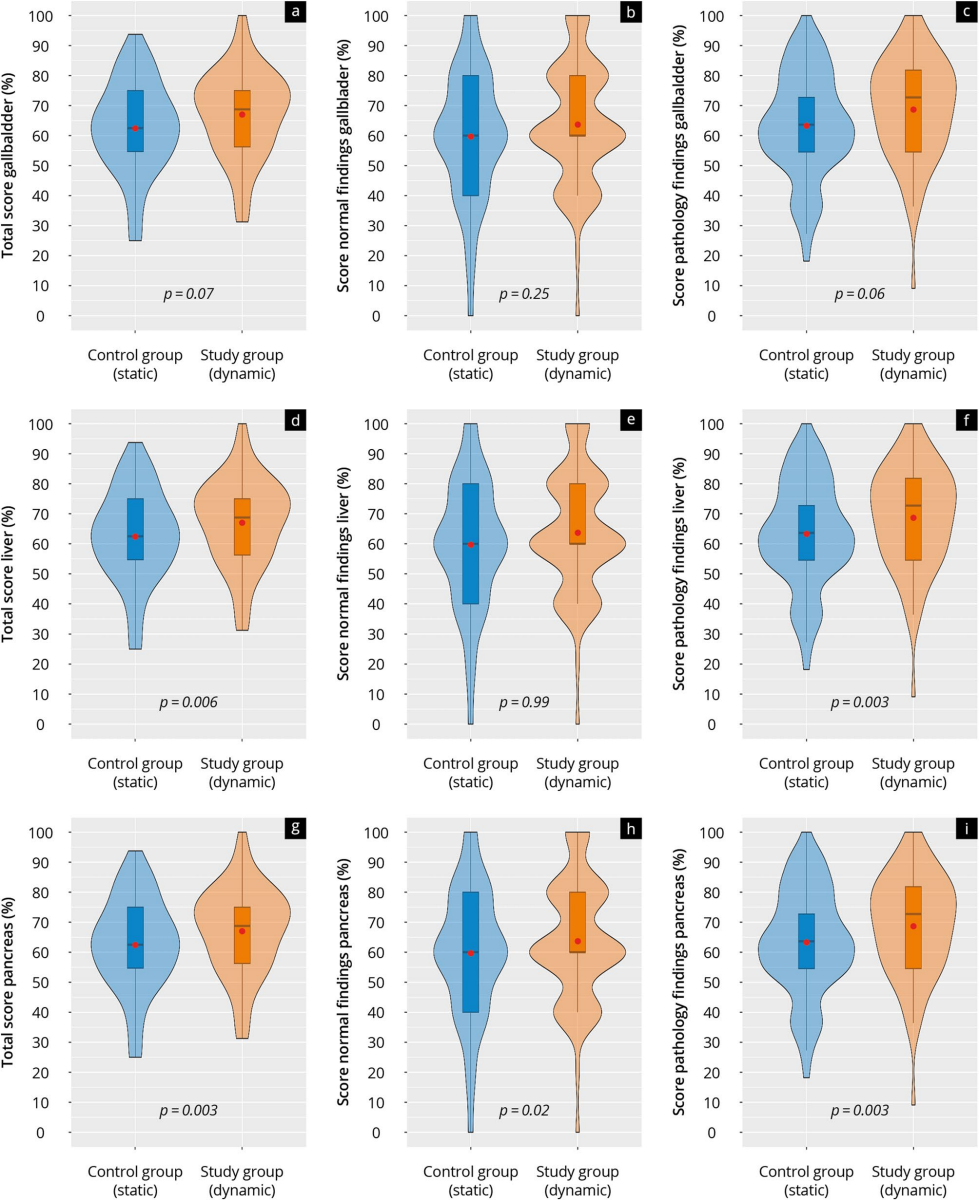
Violin plot representation of the results of the theory test of the control group (‘static’) and study group (‘dynamic’) in the (**a**–**c**) gallbladder, (**d**–**f**) liver, and (**g**–**i**) pancreas subcategories. The red dot marks the mean value

The results of the theory test are presented in Figs. 4 and 5 and Supplement 3. The study group achieved significantly higher scores in the overall theory test (study: 34.1 ± 6.3 vs. control: 30.7 ± 6.1, *p* = 0.001) and the total score of pathology findings (study: 23.5 ± 4.7 vs. control: 20.8 ± 4.6, *p* < 0.001). No significant differences were observed in the total score of normal findings (*p* = 0.08). These trends were also reflected in the liver and pancreas subcategories, but not in the gallbladder subcategory (Fig. 5).

Both groups performed generally better on normal findings than on pathology findings, with significant differences observed in some subcategories. For instance, both groups performed significantly better in “liver normal findings” compared to “liver pathology findings” (*p* < 0.01).

### Influencing factors and subgroup analysis

**Fig. 6:**
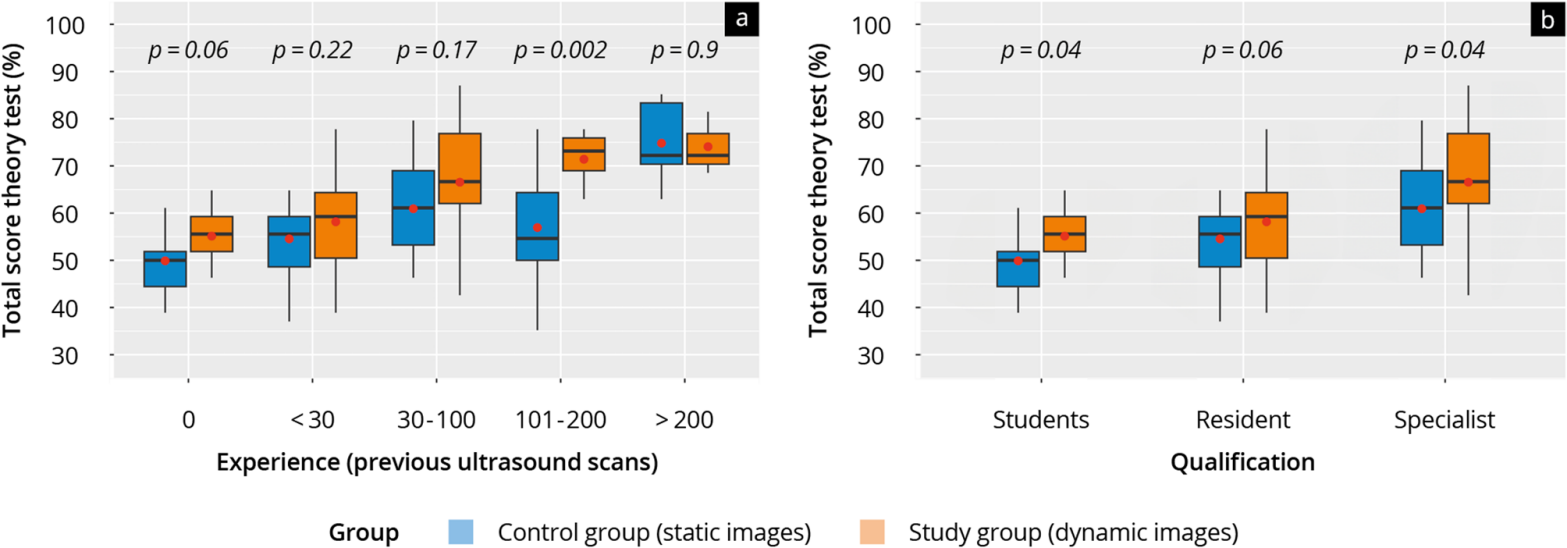
Boxplot representation of the results of the theory test of the control group (‘static’) and study group (‘dynamic’) of (**a**) different levels of experience and (**b**) qualification. The red dot marks the mean value

Membership of the study group (*p* < 0.01), a number of > 30 independent ultrasound examinations (*p* < 0.01), and current employment in internal medicine (*p* = 0.01) had a significant influence on both the total score theory test and the competence area total score pathology findings, but not on the competence area total score normal finding. Figure 6 and Supplement 4 also show the results of the theory test of the control and study groups across different levels of experience and qualification. In this subgroup analysis, participants in the study group tended to achieve higher scores in the intergroup comparison, although there was only partial evidence of significance. In addition, participants with more experience and higher qualifications achieved better results in the theory test in congruence with the calculations of the regression model.

In the subgroup analysis focusing on internists, the study group trained with dynamic images outperformed the control group trained with static images across multiple measures. For the overall score, internists in the dynamic group achieved significantly higher results compared to the static group (*p* = 0.04). While no significant differences were observed for normal findings (*p* = 0.4), internists in the dynamic group demonstrated significantly better performance in pathological findings (*p* = 0.02).

## Discussion

### Relevance of the study and key findings

Traditionally, ultrasound education combines theoretical instruction with hands-on practice to build competency [8]. The COVID-19 pandemic highlighted the need for innovative teaching methods and materials, such as blended learning, e-learning, webinars, and simulation-based training, which are now actively discussed in the literature [12, 33–36]. However, much of the focus has been on the type of teaching medium (digital vs. analogue) and instructional methods (traditional vs. innovative), with less attention given to the presentation format of ultrasound findings. The question of how static or dynamic images influence learning in ultrasound education is critical, as answering it provides an evidence-based foundation for developing future curricula and training standards. The current lack of standardized guidelines and recommendations by national and international ultrasound societies underscores the need for further research in this area [2, 37]. For the first time, this prospective, randomised, controlled study systematically investigated the impact of either static or dynamic images on the acquisition of theoretical competencies in abdominal ultrasound. The results demonstrated that participants trained with dynamic images achieved greater theoretical competency, particularly in understanding pathology findings. In contrast, both groups showed comparable results in learning normal findings. These outcomes highlight the need to integrate dynamic images more extensively into ultrasound education. A dual approach —using static images to teach sonoanatomy and dynamic images for complex pathology presentation —could enhance the efficiency of ultrasound training.

### Static vs. dynamic images in ultrasound education

Assessing the effectiveness of static versus dynamic images in ultrasound education requires evaluation of the potential advantages and disadvantages of each modality.

Static images allow learners to focus on specific details, precise measurements, and characteristic features of pathologies [2, 21, 23, 27]. This format is particularly well-suited for teaching static features, such as gallbladder wall thickness or basic sonoanatomy [20, 23, 24]. In this study, the comparable performance between groups on the “total score: normal findings” and “gallbladder pathology finding score” subcategories could be attributed to these strengths. For beginners, static images provide a structured and comprehensible learning environment with minimal cognitive load, as they are not distracted by dynamic movements [20, 21, 23, 24, 27, 38]. Additionally, static images are less resource-intensive to produce and store, making them ideal for inclusion in textbooks, scripts, and digital teaching materials [15].

The primary limitation of static images is their inability to depict real-time dynamics [20, 23, 24, 27]. They cannot represent temporal or functional processes, such as lung movement, bowel motility, or the respiratory shift of liver borders. As a result, they do not fully reflect the reality of clinical ultrasound practice [23, 24]. Complex pathologies, such as tumour infiltration or vascular abnormalities, may also be difficult to comprehend without the additional information provided by dynamic imaging [39, 40]. These limitations likely contributed to the inferior performance of the control (static image) group in the “liver pathology” and “pancreas pathology” subcategories.

Dynamic images are ideal for demonstrating functional aspects and dynamic processes, such as blood flow, organ movement, or tissue response to pressure [20, 25, 41–44]. Advantageously, dynamic images can also be integrated into digital pathology atlases [31], e-learning platforms [15, 45], or simulators [46]. Such real-time imaging is essential in fields like echocardiography and duplex sonography [47, 48] and provides practical insights into clinical applications of lung and abdominal sonography. For instance, dynamic images help to assess respiratory shift, bowel motility, or the characterisation of liver tumours using contrast agents [41–43]. This practical, real-world representation of clinical scenarios likely explains the superior performance of the dynamic group in the “total score: pathology findings” subcategory. Previous studies in musculoskeletal ultrasound and middle-ear endoscopy are in line with our results and similarly support the value of dynamic images for training [49, 50]. Another aspect that could explain the results is that dynamic images provide a clearer spatial understanding of organ or lesion structures. The sweeping motion helps visualize 3D relationships, enabling learners to recognize subtle pathologies and mentally reconstruct complex anatomical environments. This enhances intuitive comprehension and diagnostic accuracy compared to the limited perspective of static images [51].

These advantages are also consistent with findings by Nicholls et al. [52] who identified core psychomotor and cognitive skills essential for ultrasound scanning—particularly the integration of visuomotor coordination and spatial awareness. Dynamic images directly support the development of these competencies by presenting movement, anatomical variation, and probe handling in a clinically realistic manner. The improved test performance for pathology recognition in our dynamic group reflects this alignment between instructional design and skill acquisition.

However, dynamic images also have limitations, including increased cognitive load and learner difficulty in analysing details [20, 23, 24]. For beginners, the movement and abundance of information disclosed in dynamic images may be overwhelming, potentially hindering learning outcomes [20, 21, 27, 53]. Identifying individual structures or static features within dynamic images can also be challenging, thus complicating detailed analysis [23]. However, such effects were not evident in the present study. Dynamic images require more storage space, higher-performance devices, and greater bandwidth for digital teaching or documentation. However, modern database structures increasingly accommodate large data volumes [54]. For documentation and presentation of findings, both modalities should be considered to capture both static measurements and dynamic organ findings [2, 55].

Artificial intelligence could play a significant role in managing, optimising, and even generating static or dynamic images [56], as well as supporting the analysis process for learners [57]. Advanced ultrasound technologies now enable 3D imaging of organ systems and vascular structures, which has previously been recommended for integration into ultrasound education to modernise training programs further [58].

### Strengths and limitations

The randomized controlled design ensured high internal validity, while the standardized video training sessions minimized bias by controlling for content and duration across groups. The large sample size and subgroup analyses further strengthen the findings, confirming the robustness of the results. Additionally, the study’s focus on both normal findings and pathological conditions provides a comprehensive assessment of theoretical ultrasound competencies. Despite its strengths, the study has several limitations. The findings of this study are possibly limited by the study’s focus on a single area of sonography, which restricts the generalisability of the results. Additionally, the use of cross-sectional data prevents the assessment of long-term learning effects [59]. Our additional subgroup analysis demonstrates that the superior performance of participants trained with dynamic images holds true within the internist subgroup. This finding reinforces the robustness of our results and suggests that the advantage of dynamic images is not solely due to unequal professional group distribution. However, the higher proportion of internists in the study group remains a potential confounding factor and is noted as a limitation. While linear regression analysis and subsequent subgroup analysis accounted for participants’ prior experience and related professional background, other personal factors that may have influenced the test outcomes cannot be ruled out. This includes a potential selection bias due to the voluntary participation of individuals with a particular interest in ultrasound training.

## Conclusion

In summary, educators’ use of static or dynamic images in ultrasound training should be guided by specific learning objectives. Static images are particularly effective for teaching fundamental concepts and detailed sonoanatomy, whereas dynamic images excel in illustrating dynamic processes and complex pathologies. A combined approach that integrates both formats offers the greatest educational benefit by merging the precision of static images with the realism of dynamic images. Such dual strategies can optimise learning outcomes and enhance the overall effectiveness of ultrasound education. Since current training guidelines by professional societies do not explicitly address the modality of ultrasound findings used in instruction, these recommendations should be refined to provide clearer guidance on the appropriate use of imaging modalities.

## Electronic supplementary material

Below is the link to the electronic supplementary material.

Supplementary Material 1

Supplementary Material 2

Supplementary Material 3

Supplementary Material 4

## Data Availability

Data cannot be shared publicly because of institutional and national data policy restrictions imposed by the Ethics committee since the data contain potentially identifying study participants’ information. Data are available upon request from the Johannes Gutenberg University Mainz Medical Center (contact via weimer@uni-mainz.de) for researchers who meet the criteria for access to confidential data (please provide the manuscript title with your inquiry).
