# Supplementary material for: Prospective comparison of static versus dynamic images in abdominal ultrasound education - a randomised controlled trial
Source: BMC Med Educ. 2025 Jul 23;25:1102. doi: 10.1186/s12909-025-07711-9 (PMC12285136; doi:10.1186/s12909-025-07711-9)
Supplement: Supplementary file 1 — Supplementary Material 1 [file 12909_2025_7711_MOESM1_ESM.pdf]

Supplement 1 Multiple choice questions and possible answers to the different themes

**Topic**	**Gallbladder**	**Hepatic Lesion**	**Pancreatic Tumor**
Question	Which findings can you identify?	Which findings can you identify?	Which findings can you identify?
Answer A	Normal finding	Normal finding, no lesion	Normal finding
Answer B	Acute calculus cholecystitis	Hypoechoic lesion	Tumor in pancreatic head
Answer C	Sludge without inflammation	Isoechoic lesion	Tumor in pancreatic body
Answer D	Calculi without inflammation	Hyperechoic lesion	Abstention: I am uncertain
