# Supplementary material for: Prospective comparison of static versus dynamic images in abdominal ultrasound education - a randomised controlled trial
Source: BMC Med Educ. 2025 Jul 23;25:1102. doi: 10.1186/s12909-025-07711-9 (PMC12285136; doi:10.1186/s12909-025-07711-9)
Supplement: Supplementary file 2 — Supplementary Material 2 [file 12909_2025_7711_MOESM2_ESM.pdf]

Supplement 2 Baselinecharacteristics of the control group and study group; due to rounding to whole per cent not all values add up to 100%

	**Control group („Static“) N=76**	**Study group („Dynamic“) N=69**	**p-value**
	**N (%)**	**N (%)**	
**Medical Qualification**			0.69
Student	19 (25)	13 (19)	
Resident	48 (63)	47 (68)	
Specialist	8 (11)	8 (12)	
Senior Physician	4 (5)	2 (3)	
**Current Workplace**			0.12
Medical care center/ Private Practice	8 (11)	4 (6)	
Hospital	47 (62)	54 (78)	
Non-clinical	14 (18)	9 (13)	
Other	8 (11)	2 (3)	
**Current Specialty**			0.03
General Medicine	15 (20)	3 (4)	
Internal Medicine/Pediatrics	33 (43)	38 (55)	
Surgery	8 (11)	12 (17)	
Radiology	0	0	
Other	20 (26)	16 (23)	
**Ultrasound Experience**			0.74
0 examinations	17 (22)	13 (19)	
<30 examinations	22 (29)	22 (32)	
30-100 examinations	20 (26)	15 (22)	
100-200 examinations	12 (16)	16 (23)	
>200 examinations	5 (7)	3 (4)	
