# Supplementary material for: Prospective comparison of static versus dynamic images in abdominal ultrasound education - a randomised controlled trial
Source: BMC Med Educ. 2025 Jul 23;25:1102. doi: 10.1186/s12909-025-07711-9 (PMC12285136; doi:10.1186/s12909-025-07711-9)
Supplement: Supplementary file 3 — Supplementary Material 3 [file 12909_2025_7711_MOESM3_ESM.pdf]

Supplement 3 Theory Test Results

	**Control group („Static") N=76**	**Study group („Dynamic") N=69**	**p-value**
	Mean ±SD (%)	Mean ±SD (%)	
Overall test result (54 P)	30.7±6.1 (57 ± 11)	34.1 ± 6.3 (63 ±12)	0.001
Overall result of normal findings (16 P)	9.9±2.7 (62 ± 17)	10.7 ± 2.3 (67 ± 17)	0.08
Overall result of pathological findings (38 P)	20.8 ± 4.6 (55 ± 12)	23.5 ± 4.7 (62 ± 12)	< 0.001
Overall result gallbladder (16 P)	10.0 ± 2.5 (63 ± 15)	10.7 ± 2.3 (67 ± 15)	0.07
Normal findings gallbladder (5 P)	3.0 ±1.1 (60 ± 22)	3.2 ± 1.0 (64 ± 20)	0.25
Pathology gallbladder (11 P)	7.0 ± 1.9 (64 ± 18)	7.6 ± 1.9 (69 ± 17)	0.06
Overall result liver (20 P)	12.0 ± 2.8 (60 ± 14)	13.3 ± 2.5 (66 ± 13)	0.006
Normal findings liver (5 P)	4.0 ± 0.9 (79 ± 18)	4.0 ± 1.2 (79 ± 25)	0.99
Liver pathology (15 P)	8.1 ± 2.4 (54 ± 16)	9.3 ± 2.2 (62 ± 15)	0.003
Overall result pancreas (18 P)	8.7 ± 2.8 (48 ± 15)	10.1 ± 3.1 (56 ± 17)	0.003
Normal findings pancreas (6 P)	3.0 ± 1.5 (49 ± 25)	3.6 ± 1.4 (59 ± 23)	0.02
Pancreatic pathology (12 P)	5.6 ±2.0 (47 ± 17)	6.6 ± 2.1 (55 ± 18)	0.007
