# Supplementary material for: Prospective comparison of static versus dynamic images in abdominal ultrasound education - a randomised controlled trial
Source: BMC Med Educ. 2025 Jul 23;25:1102. doi: 10.1186/s12909-025-07711-9 (PMC12285136; doi:10.1186/s12909-025-07711-9)
Supplement: Supplementary file 4 — Supplementary Material 4 [file 12909_2025_7711_MOESM4_ESM.pdf]

Supplement 4 Theory test results of the control group and study group in relation to previous experience and qualifications and individual test areas; * Calculation not possible due to small subgroup

**Previous ultrasound experience (number of examinations)**			
	Control group<br>Total score theory<br>Mean ± SD in %	Study group<br>Total score theory<br>Mean ± SD in %	p-value
0 examinations	50 ± 9	55 ± 6	0.06
<30 examinations	55 ± 9	58 ± 10	0.22
30-100 examinations	61 ± 10	67 ± 13	0.17
100-200 examinations	57 ± 12	71 ± 9	0.002
>200 examinations	75 ± 9	74 ± 7	0.9
	Control group<br>Total score normal findings<br>Mean ± SD in %	Study group<br>Total score normal findings<br>Mean ± SD in %	p-value
0 examinations	54 ± 14	55 ± 10	0.84
<30 examinations	63 ± 16	65 ± 17	0.58
30-100 examinations	66 ± 14	68 ± 17	0.77
100-200 examinations	59 ± 23	77 ± 15	0.03
>200 examinations	78 ± 10	75 ± 17	0.83
	Control group<br>Total score pathology findings<br>Mean ± SD in %	Study group<br>Total score pathology findings<br>Mean ± SD in %	p-value
0 examinations	48 ± 10	55 ± 7	0.03
<30 examinations	51 ± 10	56 ± 11	0.13
30-100 examinations	59 ± 12	66 ± 14	0.14
100-200 examinations	56 ± 10	69 ± 10	0.003
>200 examinations	73 ± 10	75 ± 10	0.86
**Qualification**			
	Control group<br>Total score theory<br>Mean ± SD in %	Study group<br>Total score theory<br>Mean ± SD in %	p-value
Student	50 ± 6	55 ± 6	0.04
Resident	58 ± 11	64 ± 12	0.006
Specialist	60 ± 10	71 ± 10	0.04
Senior Physician	*	*	
	Control group<br>Total score normal findings<br>Mean ± SD in %	Study group<br>Total score normal findings<br>Mean ± SD in %	p-value
Student	52 ± 16	54 ± 12	0.73
Resident	63 ± 16	70 ± 16	0.05
Specialist	67 ± 13	72 ± 16	0.53
Senior Physician	*	*	
	Control group<br>Total score pathology findings<br>Mean ± SD in %	Study group<br>Total score pathology findings<br>Mean ± SD in %	p-value
Student	49 ± 7	55 ± 8	0.03
Resident	55 ± 12	62 ± 13	0.006
Specialist	57 ± 11	69 ± 13	0.05
Senior Physician	*	*	
